# Obtaining full contact for measuring polydimethylsiloxane mechanical properties with flat punch nanoindentation

**DOI:** 10.1016/j.mex.2015.09.004

**Published:** 2015-10-09

**Authors:** Federico De Paoli, Alex A. Volinsky

**Affiliations:** Department of Mechanical Engineering, University of South Florida, Tampa, FL 33620, USA

**Keywords:** Flat punch full contact nanoindentation, Elastic modulus, Flat punch, Full contact, Nanoindentation, Loading slope, Polydimethylsiloxane, Mechanical properties

## Abstract

Procedure to establish full contact between the sample and the 1 mm diameter cylindrical flat punch tip to measure polydimethylsiloxane (PDMS) mechanical properties using the Hysitron TriboIndenter is described. This procedure differs from the standard automated indentation because each indent has to be performed manually after establishing full contact with the sample surface. Incomplete contact happens because of the sample tilt with respect to the flat punch surface and results in incorrect elastic modulus values.

• Automated indentation results in incorrect values of the elastic modulus due to initial incomplete contact between the flat punch and the PDMS sample surface, caused by the tilt, and using the unloading slope, which is affected by viscoelastic soft polymer deformation.
• Correct procedure requires establishing the full contact between the tip and the sample. This is achieved by moving the tip into the sample in 1–2 μm increments, up to 40–80 μm maximum combined displacement, until the loading stiffness no longer increases.
• The elastic modulus is calculated from the loading stiffness and the diameter of the flat punch, instead of the unloading stiffness, which is larger due to viscoelastic unloading. After establishing the full contact, other mechanical testing can be conducted.

## Graphical abstract

**Figure:**
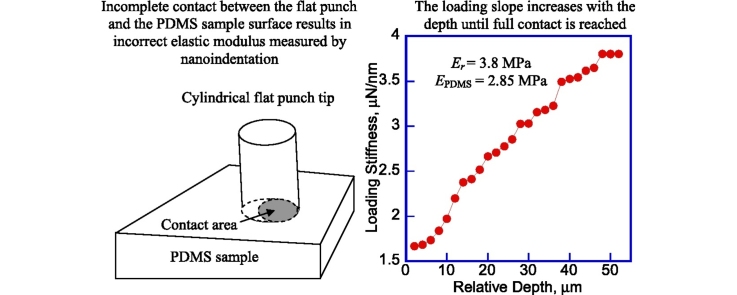

## Method details

**Fig. 1:**
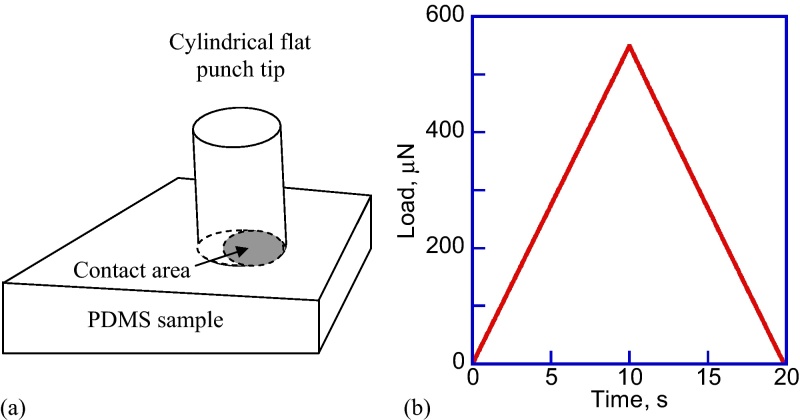
(a) Schematic of the flat punch tilt with respect to the sample surface; (b) load function (load vs. time) for indentations conducted in this experiment.

A method to properly conduct flat punch nanoindentation experiments with polydimethylsiloxane (PDMS) samples and calculate their elastic modulus is described. Hysitron TriboIndenter (Hysitron, USA), equipped with a 1 mm diameter flat cylindrical punch tip and a standard transducer, was used. Calculating the elastic modulus of the samples depends on the contact area between the tip and the sample, which should not change during the flat punch indentation. However, the full contact between the flat punch tip and the sample is not initially established due to the unavoidable sample tilt with respect to the cylindrical flat punch surface, which results in incorrect elastic modulus values when using automated indentation [1], [2]. This situation is shown schematically in Fig. 1(a), and applies for the larger diameter flat punch cylindrical tips. While indentation experiments using flat punch indentation of PDMS have been conducted before [2], [3], there is no study explicitly addressing the problem of full contact.

Every sample tested is described with the acronym PDMS, followed by the elastomer base/curing agent ratio. For example, one of the samples studied is named PDMS 10:1, meaning that the base/agent ratio is 10, with 10 mass units of the silicone elastomer base mixed with 1 unit mass of the silicone elastomer curing agent. The article describes experiments conducted with two different PDMS 10:1 samples, however other ratios were also tested using the same method, namely PDMS 30:1 and PDMS 50:1. The testing method does not change with different samples.

To start the procedure it is necessary to first set up the Hysitron TriboIndenter and the TriboScan software. The machine needs to be calibrated, which is achieved following the standard air indentation procedure [4]. The air indentation is performed to calibrate the transducer electrostatic force and check the transducer plates spacing [4], [5]. This is the only step that resembles the so-called “standard” indentation test. The rest of the procedure is different, which represents the essence of this paper.

The experiment consists of moving the tip deeper into the sample surface in 1–2 μm increments relative to the previous position by manually raising the sample stage. The relative sample stage movement increment is chosen as 1–2 μm, or higher each time, based on the maximum displacement reached in the previous indent at the pre-defined 600 μN maximum load. However, attention should be paid not to exceed the total displacement above 40–50 μm. This limitation is due to the spacing between the transducer capacitor plates of around 80–100 μm. Assuming that the initial position is at the centre of the available displacement range, it is safe to keep the total stage movement under 40–50 μm. The total allowable displacement also depends on the sample stiffness, and it is safe to have up to 80 μm maximum displacement for the softer PDMS samples.

Sample safety boundaries do not need to be defined using this modified testing procedure. Before starting the test, the tip is moved manually, using the stage, above the sample surface and lowered to make the first contact with the PDMS sample. The problem with this kind of material is that even if the tip is in contact with the sample, there is no guarantee that it is in full contact, since the sample surface is always tilted with respect to the flat punch tip surface, as shown schematically in Fig. 1(a). Before collecting any meaningful indentation data, full contact between the tip and the sample surfaces must be established. If the full contact is not established, erroneous modulus values will result.

The tip has to be moved deeper into the sample, until the measured stiffness of the sample would not change with further increase of the relative indentation depth. The load function chosen in this experiment is shown in Fig. 1(b). The peak load value is 600 μN, while the loading time of 10 s is the same as the unloading time. This typical maximum load is used during the air indentation calibration, which results in about 4–5 μm tip travel range in air, without contacting the sample [4]. The Hysitron TriboIndenter transducer has ∼5 μm maximum travel range, thus, the selected 600 μN maximum load will not result in exceeding the maximum transducer displacement. The indents are performed in the open-loop mode, i.e. pseudo-load control.

**Fig. 2:**
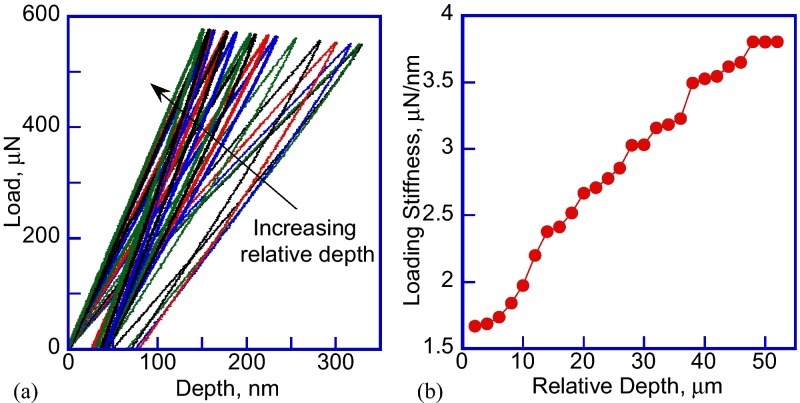
(a) Load–displacement curves showing increasing stiffness of the loading slope with increasing relative depth; (b) loading stiffness vs. relative depth to establish full contact between the flat punch tip and the sample surfaces.

**Fig. 3:**
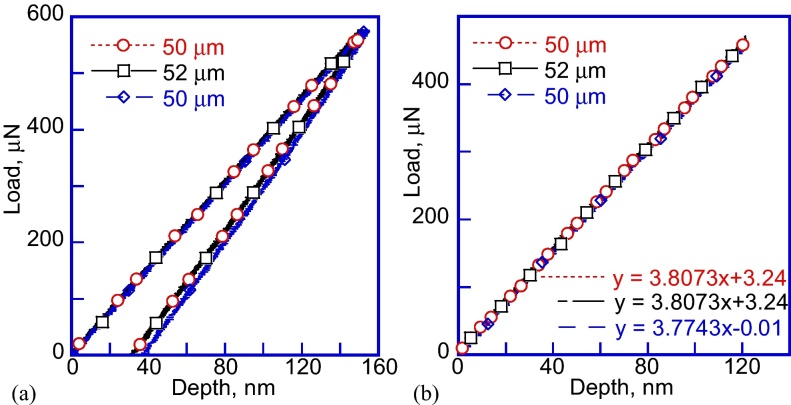
(a) Load–displacement curves after reaching full contact and loading slope saturation at 50 μm relative tip depth; (b) loading portions of the load–displacement curves at 50 μm and 52 μm relative tip depth with the corresponding linear fits.

The method to conduct the flat punch nanoindentation experiments and calculate the elastic modulus of the PDMS samples consists of the following steps:

1) The sample is mounted on the stage using standard suggested procedure [4]. Air indentation calibration is performed to make sure that the Hysitron transducer is calibrated and working properly. The sample stage is moved to position the tip above the sample surface, and then it is moved along the vertical Z direction until the first contact between the tip and the sample is established. In case the safety load is exceeded, one can press the “AUTO ZERO” button on the transducer controller. While this does not happen during every test, reaching the safety load stops the stage motors, which can be avoided. The authors contacted the nanoindenter equipment manufacturer to establish this procedure.
2) The first indentation is performed into the PDMS sample, however the loading slope results are not consistent, and change as a function of the relative indentation depth due to incomplete contact between the flat punch tip and the PDMS sample, as seen in Fig. 2(a). The sample stage is manually moved up using the Triboscan software controls towards the tip to increase the relative indentation depth in 1–2 μm increments, which results in increased loading stiffness in Fig. 2(b). The stage movement increment is based on the maximum transducer 5 μm displacement range and the maximum indentation depth of the previous indent, which was on the order of 300 nm in Fig. 2(a). This procedure is repeated until the loading slope doesn’t increase anymore. In this case the procedure was repeated 24 times before obtaining a consistent result in Fig. 2(b).
3) After moving the sample stage by 48 μm into the indenter tip, there was no further change in the loading stiffness in Fig. 2(b) with the stage motion of 50–52 μm. This proves that the full contact between the tip and the PDMS sample is reached. Decreasing the depth by 2 μm also demonstrates that there is full contact, since the loading stiffness of the three load–displacement curves is the same in Fig. 3(a). Similar flat punch indentation depth of ∼50 μm was used by other researchers [5].
4) In Fig. 2(b) it is clear that even at 50 μm and 52 μm relative displacements there is no change of the loading slope. If the tip is retracted by 2 μm, therefore returning to the 50 μm relative depth, the loading slope remains the same in Fig. 3(b). This is the proof that the full contact is established. Unchanged loading slope also suggests elastic contact and no plastic deformation of the PDMS sample. These tests were performed using two different PDMS 10:1 samples, 10 mm and 5 mm thick. PDMS 30:1 and 50:1 with similar thickness of 5 mm were also tested, however the data are not presented here.
5) After isolating the loading slope in Fig. 3(b), it is possible to calculate the reduced elastic modulus of the PDMS sample by using the loading slope and the following formula: $E_{r}=S/D$ [5]. Here, *S* is the measured loading stiffness, and *D* is the flat punch tip diameter. The reduced elastic modulus of the PDMS 10:1 sample in this study is 3.8 MPa. The obtained result can be considered acceptable, since it is of the same order of magnitude as the values measured using different techniques described in the literature [5], [7], [9]. The elastic modulus of the PDMS is 25% smaller than the reduced modulus, and is consistent with previous measurements: *E*_PDMS_ = 0.75, *E*_*r*_ = 2.85 MPa [5], [6], [7], [8], [9].

Metals and other ductile materials undergo elastic and plastic deformation during loading, followed by elastic recovery upon the unloading. This is why the unloading slope is used to measure the stiffness and calculate the elastic modulus of these materials. However, viscoelastic soft polymers are different, since they undergo viscoelastic deformation upon the unloading. This is why the slope during fast loading is utilized in the described method to calculate the elastic properties of soft PDMS. Loading time should be reduced to minimize the effects of viscoelastic deformation during loading.

This experiment has been conducted on two different PDMS 10:1 samples multiple times. Additionally, PDMS 30:1 and 50:1 samples were also tested using the described method. The PDMS 10:1 samples were created at different times. Significantly older PDMS 10:1 samples (over 5 years old) have a higher elastic modulus, demonstrating some ageing phenomena. Once the full contact between the cylindrical flat punch tip and the PDMS sample surface is established, other experiments can be performed, including stress relaxation and dynamic testing [5], [7]. When conducting flat punch nanoindentation experiments one has to make sure there is full contact between the tip and the sample surface and use the loading stiffness for viscoelastic soft polymers to obtain accurate and meaningful results [5], [6], [7], [8], [9], [10].
